# Randomised trial of trastuzumab deruxtecan and biology-driven selection of neoadjuvant treatment for HER2-positive breast cancer: a study protocol of ARIADNE

**DOI:** 10.1136/bmjopen-2025-102626

**Published:** 2025-08-27

**Authors:** Alexios Matikas, Bjørn Naume, Hans Wildiers, Gabe Sonke, Maria Vittoria Dieci, Andreas Karakatsanis, Anne Andersson, Elin Barnekow, Luisa Edman Kessler, Zakaria Einbeigi, Fredrika Killander, Barbro Linderholm, Aglaia Schiza, Antonis Valachis, Andreas Nearchou, Olav Engebraaten, Alina Porojnicu, Mari Hiorth Soland, Bård Mannsåker, Sunil X Raj, Egil Støre Blix, Cecilie Soma Nordstrand, Matteo Lambertini, Claudio Vernieri, Kevin Punie, Christos Sotiriou, Jonas Bergh, Guillermo Villacampa, Athanasios Zouzos, Mats Hellström, Johan Hartman, Theodoros Foukakis

**Affiliations:** 1 Breast Center, Theme Cancer, Karolinska University Hospital and Karolinska Comprehensive Cancer Center, Stockholm, Sweden; 2 Department of Oncology/Pathology, Karolinska Institutet, Stockholm, Sweden; 3 Department of Oncology, Oslo University Hospital, Oslo, Norway; 4 Department of General Medical Oncology and Multidisciplinary Breast Centre, University Hospitals Leuven, Leuven, Belgium; 5 Division of Medical Oncology, Netherlands Cancer Institute, Amsterdam, The Netherlands; 6 Dipartimento di Scienze Chirurgiche, Oncologiche e Gastroenterologiche, Università di Padova, Padova, Italy; 7 Oncologia 2, Istituto Oncologico Veneto IRCCS, Padova, Italy; 8 Department of Surgical Sciences, Uppsala University, Uppsala, Sweden; 9 Section for Breast Surgery, Uppsala University Hospital (Akademiska), Uppsala, Sweden; 10 Department of diagnostics and intervention, oncology, Umeå University, Umeå, Sweden; 11 Department of Clinical Science and Education, Södersjukhuset, Karolinska Institutet, Stockholm, Sweden; 12 Department of Oncology, Södersjukhuset, Stockholm, Sweden; 13 Breast Center, Capio Saint Göran’s Hospital, Stockholm, Sweden; 14 Department of Oncology, Institute of Clinical Sciences, Sahlgrenska Academy, University of Gothenburg, Gothenburg, Sweden; 15 Department of Medicine and Oncology, Southern Älvsborg Hospital, Borås, Sweden; 16 Division of Oncology and Pathology, Department of Clinical Sciences Lund, Faculty of Medicine, Skåne University Hospital, Lund University, Lund, Sweden; 17 Institution of Clinical Sciences, Department of Oncology, Sahlgrenska Academy at Gothenburg University, Gothenburg, Sweden; 18 Department of Oncology, Uppsala University Hospital and department of Immunology, Genetics and Pathology Uppsala University, Uppsala, Sweden; 19 Department of Oncology, Faculty of Medicine and Health, Örebro University, Örebro, Sweden; 20 Department of Oncology, Mälarsjukhuset, Eskilstuna, Sweden; 21 Department of Oncology, Drammen Hospital, Vestre Viken Hospital Trust, Drammen, Norway; 22 Department of Oncology, Stavanger University Hospital, Stavanger, Norway; 23 Department of Oncology, Nordland Hospital Trust, Bodø, Norway; 24 Breast Cancer Unit, Cancer Clinic, University Hospital in Trondheim, Trondheim, Norway; 25 Department of Oncology, University Hospital of North Norway, Tromsø, Norway; 26 Department of Oncology, Møre og Romsdal Hospital Trust, Alesund, Norway; 27 Department of Medical Oncology, U.O. Clinica di Oncologia Medica, IRCCS Ospedale Policlinico San Martino, Genova, Italy; 28 Department of Internal Medicine and Medical Specialties (DIMI), School of Medicine, University of Genova, Genova, Italy; 29 Medical Oncology Department, Breast Unit, Fondazione IRCCS Istituto Nazionale dei Tumori, Milano, Italy; 30 Oncology and Hematology-Oncology Department, University of Milan, Milan, Italy; 31 Department of Medical Oncology, Ziekenhuis Aan de Stroom, Wilrijk, Belgium; 32 Breast Cancer Translational Research Laboratory, Institut Jules Bordet, Hôpital Universitaire de Bruxelles, Université Libre de Bruxelles, Brussels, Belgium; 33 SOLTI Cancer Research Group, Barcelona, Spain; 34 Statistics Unit, Vall d'Hebron Institute of Oncology (VHIO), Barcelona, Spain; 35 Department of Clinical Pathology and Cancer Diagnostics, Karolinska University Hospital, Stockholm, Sweden

**Keywords:** Breast tumours, Clinical trials, Drug Therapy

## Abstract

**Introduction:**

Neoadjuvant therapy is the standard of care for the treatment of human epidermal growth factor receptor 2 (HER2)-positive breast cancer (BC). Studies on first-generation antibody-drug conjugates, such as trastuzumab emtansine (T-DM1), showed equal or slightly lower efficacy than chemotherapy combined with dual HER2 blockade. Trastuzumab deruxtecan (T-DXd) is a next-generation conjugate approved for the treatment of metastatic HER2-positive and HER2-low BC, with greatly improved efficacy compared to T-DM1.

**Methods and analysis:**

ARIADNE is an academic, international, open-label, randomised, comparative phase IIB trial. A total of 370 patients with non-metastatic HER2-positive BC and an indication for neoadjuvant therapy will be included and randomised 1:1 to receive either (1) docetaxel (or paclitaxel), carboplatin, trastuzumab (H) and pertuzumab (P) for three cycles or (2) T-DXd for three cycles. Further treatment is based on the intrinsic molecular subtype determined by the Prosigna assay: patients with HER2-enriched disease (estimated 60%) continue the same treatment for three more cycles. Patients with oestrogen receptor (ER)-positive and luminal (estimated 30%) disease receive H and P for three cycles, combined with letrozole and ribociclib for two cycles. Patients with ER-negative and luminal, basal-like or normal-like disease (estimated 10%) either continue the same treatment for three more cycles in the case of a radiologic complete response, or, in the case of no complete response, they receive four cycles of dose-dense epirubicin and cyclophosphamide. The primary endpoint of ARIADNE is locally assessed rate of pathologic complete response in the molecularly HER2-enriched population, defined as ypT0/Tis, ypN0, as determined by a pathologist blinded to treatment assignment (intention-to-treat (ITT) analysis). Key secondary endpoints include time-to-event endpoints (event-free, recurrence-free, distant recurrence-free and overall survival), safety, health-related quality of life and translational studies.

**Ethics and dissemination:**

The study has been approved by the Swedish Medical Products Agency (Läkemedelsverket), the Swedish Ethical Review Authority (Etikprövningsmyndigheten) and the Norwegian Ethics Committee for Clinical Trials on Medicinal Products and Medical Devices, as well as by the review boards at all participating centres. Applications for ethical approval in Belgium, the Netherlands and Italy are ongoing. We intend to publish the results of the study in a scientific journal. The study results will be submitted to the European Union (EU) database within 1 year after the end of the clinical trial (CT).

**Trial registration number:**

EU CT registration number: 2022-501504-95-00; ClinicalTrials.gov identifier: NCT05900206.

**STRENGTHS AND LIMITATIONS OF THIS STUDY**

- The ARIADNE study is a randomised, multicentre trial conducted in five European countries.
- The study evaluates both a novel antibody-drug conjugate and a biology-driven approach to neoadjuvant therapy for human epidermal growth factor receptor 2-positive breast cancer.
- The study has a strong translational focus, with longitudinal tissue and plasma sampling before, during and after study treatment.
- The design is open-label, which could influence toxicity and quality of life outcomes.
- The sample size is not powered to detect small differences in long-term patient survival.

## Introduction

 Neoadjuvant chemotherapy (NACT) for early breast cancer (BC) was first introduced as an attempt to downstage locoregionally advanced tumours in order to facilitate potentially curative surgical resection. However, the recognition that pathologic complete response (pCR) in the surgical specimen is strongly prognostic in patients with human epidermal growth factor receptor 2 (HER2)-positive or triple-negative BC^1^ and that NACT is equivalent to adjuvant chemotherapy in terms of efficacy^2 3^ has led to the rapid uptake of NACT. Besides evaluating chemosensitivity in vivo, NACT has the added advantage of permitting clinical trials (CTs) with much shorter follow-up periods and smaller sample sizes compared with trials at the adjuvant setting. Moreover, the ability to serially extract tissue and to evaluate the temporal evolution of the tumour at the histologic, genomic and proteomic levels has greatly improved our understanding of the underlying tumour biology under the selection pressure applied by systemic treatment.^4^,^6^

A series of trials has established that the addition of trastuzumab (H) and pertuzumab (P) to chemotherapy significantly improves the likelihood of achieving pCR in early HER2-positive BC,^7^ which comprises approximately 15% of all BC cases. As an example, anthracycline-free regimens that combine dual blockade, a taxane and carboplatin (TCHP) result in pCR rates of around 55–65%.^8 9^ At the same time, further adjuvant treatment with trastuzumab emtansine (TDM-1) if pCR is not achieved significantly improves outcomes.^10 11^ Due to these excellent results, recent efforts have focused on de-escalation to decrease acute and long-term toxicity while not compromising outcomes. For example, in the Swedish PREDIX HER2 trial, neoadjuvant T-DM1 led to a 44.1% pCR rate, similar to that with standard docetaxel/H/P (46.4%)^12^; long-term follow-up patient outcomes were excellent and similar between the two groups.^5^

Since the time PREDIX HER2 was conducted (2014–2018), the field has moved at a fast pace. Novel antibody-drug conjugates (ADCs) such as trastuzumab deruxtecan (T-DXd) show even higher efficacy with acceptable side-effect profiles in the treatment of metastatic HER2-positive BC than what was previously achieved with T-DM1. In a direct comparison in the DESTINY-Breast03 trial, median progression-free survival with T-DXd was more than four times longer than with T-DM1 (28.8 vs 6.8 months).^13^ These highly intriguing results merit evaluation of T-DXd as neoadjuvant therapy, considering the already promising efficacy of preoperative T-DM1. In addition, two key observations provide additional rationale for ARIADNE: cyclin-dependent kinase 4/6 inhibitors, highly successful in treating metastatic luminal BC,^14^ are investigated in the neoadjuvant setting^15^ and have shown promising results for the treatment of metastatic HER2-positive BC^16^; and HER2-enriched cancers, a group that comprises approximately 60% of all HER2-positive BCs, are exquisitely sensitive to HER2 blockade.^17^

In summary, the first generation of de-escalation trials showed that T-DM1 is associated with comparable efficacy, lower risk of adverse events (AEs) and improved health-related quality of life (HRQoL) compared with standard treatment. Based on existing evidence and on the experience accumulated through the conduct of the PREDIX HER2 trial, the aim of the ARIADNE trial is to employ biology-driven stratification to deliver tailored neoadjuvant therapy with second-generation ADC or de-escalated targeted therapy.

## Methods and analysis

The reporting of this study protocol follows the Standard Protocol Items: Recommendations for Interventional Trials guidelines,^18^ which are available online as supplementary data. Information provided hereunder is based on protocol V.3.0, 29 November 2024.

### Study hypothesis and aim

The main research question of ARIADNE is whether T-DXd is more effective than standard neoadjuvant therapy with TCHP in a subgroup of molecularly selected patients. The second research question is whether there are clinicopathological, circulating, genetic and genomic biomarkers that may select candidates for a de-escalation approach. The third research question concerns the evaluation of therapy stratification according to intrinsic molecular subtype. Finally, the fourth research question is whether de-escalated therapy leads to decreased toxicity and improved quality of life during and after treatment.

The primary objective of the study is to compare, in terms of locally assessed pCR rates, standard neoadjuvant therapy versus T-DXd monotherapy in patients with molecularly HER2-enriched and clinically HER2-positive, non-metastatic BC. A full list of study objectives is presented in table 1.

**Table 1:** Study objectives of the ARIADNE trial

Primary objective	To compare, in terms of locally assessed pCR rates, standard neoadjuvant therapy versus T-DXd monotherapy in patients with molecularly HER2-enriched and clinically HER2-positive, non-metastatic BC.
Key secondary objectives	To perform translational studies on clinicopathologic factors and the tissues or peripheral blood for the discovery of prognostic or predictive biomarkers that are relevant to the treatments in the trial.
	To compare, in terms of locally assessed pCR rates, the two patient groups of the initial randomisation of molecularly unselected patients between standard and experimental therapy, regardless of administered therapy beyond cycle 3.
Other secondary objectives	To investigate the pCR rates for patients with HER2-positive but non-HER2-enriched BC, who will receive sequential combinations of T-DXd and either ribociclib combined with endocrine therapy and dual HER2-blockade for ER-positive and molecularly luminal cancers (de-escalated, chemotherapy-free regimen) or response-adjusted therapy for normal-like, basal-like or ER-negative and luminal cancers (escalated therapy). Their outcomes will be compared with historical controls from the PREDIX HER2 study and a large cohort of about 500 HER2-positive patients treated with neoadjuvant therapy at Stockholm 2007–2017.
	Efficacy measures for the comparison of standard and experimental treatment, both time-to-event (relapse-free, distant disease-free and OS) and short-term endpoints (pathologic response according to RCB, rates of complete radiologic response).
	To evaluate the effect of standard and experimental treatment on the type of surgery performed in the breast and the axilla.
	To evaluate PRO measures for HRQoL in the two treatment arms.
	To evaluate the safety and tolerability of the two treatment arms.

BC, breast cancer; ER, oestrogen receptor; HER2, human epidermal growth factor receptor 2; HRQoL, health-related quality of life; OS, overall survival; pCR, pathologic complete response; PRO, patient-reported outcome; RCB, residual cancer burden; T-DXd, trastuzumab deruxtecan.

### Study design

**Figure 1:**
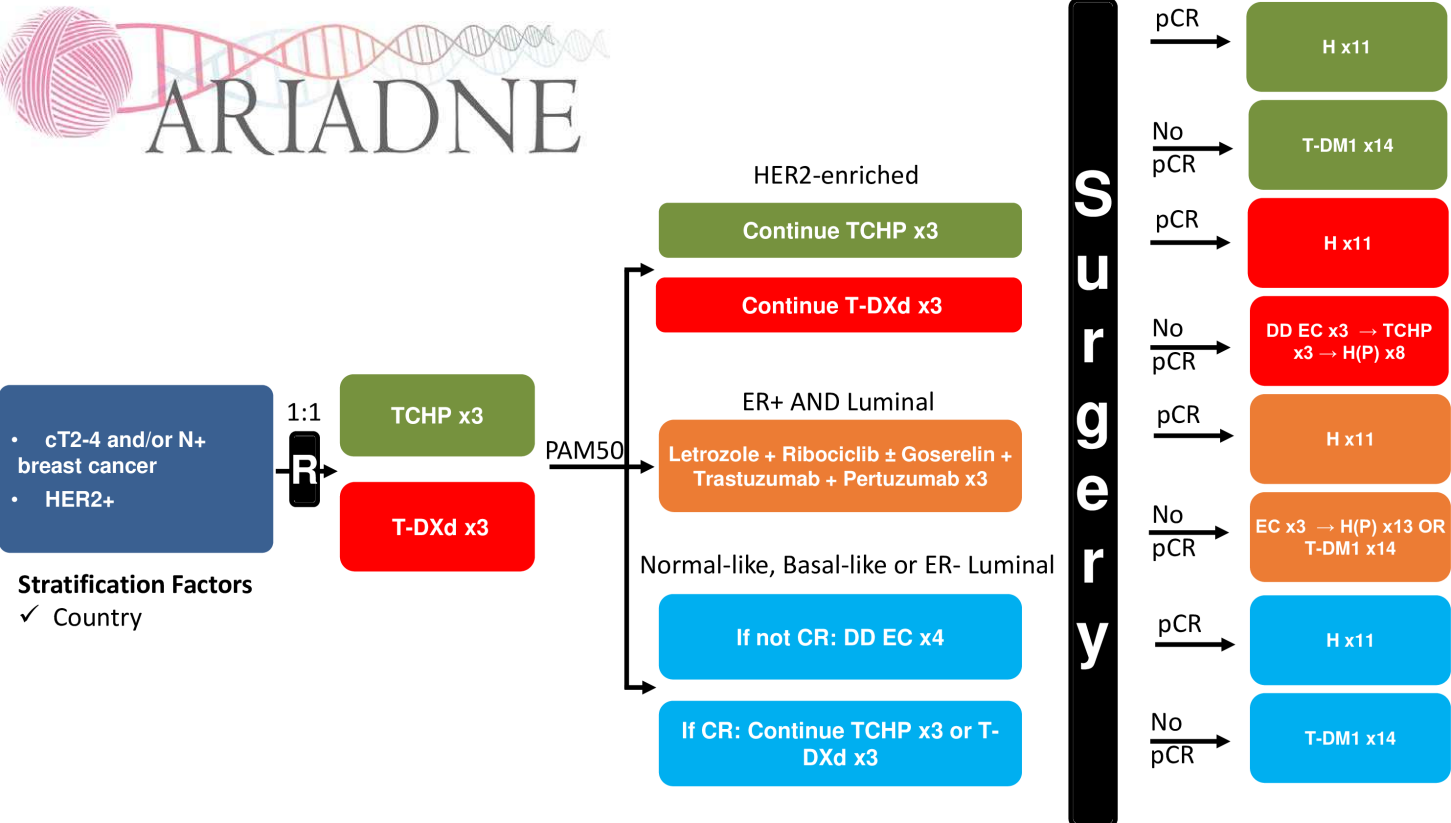
Study scheme of the ARIADNE trial. CR, complete response; cT2–4, clinical extent of primary tumour, stage T2–T4; DD, dose-dense; EC, epirubicin and cyclophosphamide; ER, oestrogen receptor; H, trastuzumab; HER2, human epidermal growth factor receptor 2; P, pertuzumab; PAM50, Prediction Analyis of Microarray 50; pCR, pathologic complete response; R, randomization; TCHP, docetaxel, carboplatin, trastuzumab, pertuzumab; T-DM1, trastuzumab emtansine and T-DXd, trastuzumab deruxtecan.

ARIADNE (European Union (EU) CTs register number: 2022-501504-95-00; ClinicalTrials.gov identifier: NCT05900206) is an international, multicentre, open-label, randomised, comparative phase IIB trial. Patients with non-metastatic HER2-positive BC are randomised 1:1 and stratified per country to receive either (1) standard neoadjuvant TCHP therapy for three cycles or (2) T-DXd for three cycles. Further treatment is based on the intrinsic molecular subtype, which will be assigned from formalin-fixed paraffin-embedded tissue, obtained from a baseline pretherapy biopsy (figure 1):

- HER2-enriched: these patients continue with the same treatment for three more cycles.
- ER-positive and luminal: these patients receive H and P for three cycles, combined with letrozole and ribociclib (plus goserelin for premenopausal patients) for two cycles.
- ER-negative and luminal, basal-like or normal-like: if the patient has attained a radiologic complete response, she/he continues with the same previously allocated treatment for three more cycles. In the case of no complete response, the patient receives four cycles of dose-dense (DD) epirubicin and cyclophosphamide (EC).^19^,^22^

Following surgery, patients are treated per the recommendations of the treating physician and in accordance with international treatment guidelines, outside the context of the trial. However, detailed treatment recommendations are provided in the study protocol, depending on the administered preoperative therapy and whether the subject attained pCR or not, as shown in figure 1.

### Setting of the study

The study sponsor is Karolinska University Hospital, Stockholm, Sweden. The study is conducted in 10 sites in Sweden (Stockholm: Karolinska University Hospital, Södersjukhuset and Saint Göran’s Hospital; Uppsala University Hospital; Örebro University Hospital; Umeå: Norrlands University Hospital; Malmö: Skåne University Hospital; Borås: Södra Älvsborg Hospital; Gothenburg: Sahlgrenska Hospital and Eskilstuna: Mälarsjukhuset) and seven sites in Norway (Oslo University Hospital, Drammen Hospital, Stavanger University Hospital, Ålesund Hospital, St Olav Hospital, Nordland Hospital and North Norway University Hospital), while during Q3 (third quarter) 2025, an additional three sites in Belgium, one in the Netherlands and three in Italy will be activated. The study opened for inclusion in Q4 2023 (first patient randomised: 26 October 2023) and has a planned 3-year inclusion period. The primary endpoint will be analysed approximately 6 months after randomisation of the last patients, during Q2 2027.

### Study participants and randomisation

Women and men aged 18 years or older, with non-metastatic HER2-positive BC with an indication for neoadjuvant therapy, are eligible for inclusion in the study. The full list of inclusion and exclusion criteria is provided in table 2. After written informed consent is obtained by authorised study investigators and all screening assessments have been performed, no more than four business days before the start of treatment, eligible patients are randomised in a 1:1 ratio between the two treatment arms. Centralised randomisation is performed at the Centre for Clinical Cancer Trials, Theme Cancer, Karolinska University Hospital, Stockholm, using a web-based procedure (TENALEA) with random permuted blocks (block sizes of 2 or 4). Randomisation is stratified by country. Neither study participants nor investigators are blinded to treatment allocation.

**Table 2:** Patient inclusion and exclusion criteria in ARIADNE

Inclusion criteria	Before patient registration/randomisation, written informed consent must be given according to ICH/GCP, and national/local regulations.
	Women or men aged 18 years or older.
	Histologically confirmed BC with an invasive component measuring >20 mm and/or with morphologically confirmed spread to regional lymph nodes (stage cT2-cT4 with any cN, or cN1-cN3 with cT1-4). Ipsilateral multifocal and multicentric tumours are allowed.
	ECOG performance status 0 or 1 at the time of randomisation.
	Known oestrogen-receptor and/or progesterone receptor status, as assessed locally by IHC. The cut-off for positivity for ER/PR for this study is at least 10% of cell nuclei staining for ER or PR, respectively.
	Known HER2-positive BC defined as an IHC status of 3+. If IHC is 2+, a positive in situ hybridisation (FISH, CISH or SISH) test is required by local laboratory testing. ISH positivity is defined as a ratio of ≥2 for the number of HER2 gene copies to the number of signals for chromosome 17 copies.
	LVEF≥50% within 28 days before randomisation.
	Adequate bone-marrow, hepatic and renal function defined as laboratory tests within 7 days prior to enrolment: Haematology: Absolute granulocytes>1.5×10^9^/L. Platelets>100×10^9^/L. Hb>90 g/L. Biochemistry Bilirubin≤ULN. Serum creatinine≤1.5×ULN. AST and ALT≤1.5×ULN. Albumin≥30 g/L. Coagulation: INR/PT≤1.5×ULN, unless the subject is receiving anticoagulant therapy and INR/PT is within intended therapeutic range. aPTT≤1.5×ULN, unless the subject is receiving anticoagulant therapy and aPTT is within intended therapeutic range.
	Availability of tumour and blood samples as described in the protocol.
	Negative serum pregnancy test for women of childbearing potential or for patients who have experienced menopause onset <12 months prior to randomisation. Patients of childbearing potential must be willing to use one highly effective contraception or two effective forms of non-hormonal contraception.
	Participants must be able to communicate with the investigator and comply with the requirements of the study procedures.
Exclusion Criteria	Participation in other interventional trials.
	Known presence of distant metastases, including node metastases in the contralateral thoracic region or the mediastinum. Synchronous contralateral BC is allowed if the tumour is HER2-positive.
	Other malignancies diagnosed during the past 5 years, except adequately controlled, limited basal cell carcinoma or squamous-cell carcinoma of the skin, in situ melanoma or carcinoma in situ of the cervix.
	History of invasive BC.
	History of DCIS, except for patients treated exclusively with mastectomy >5 years prior to diagnosis of current BC.
	Active cardiac disease or a history of cardiac dysfunction, including any of the following: History of unstable angina pectoris, myocardial infarction or recent (<6 months) cardiovascular event, including stroke and pericarditis. History of documented congestive heart failure (New York Heart Association Functional Classification II–IV). Documented cardiomyopathy. QTc >450 ms as measured by Fridericia’s formula, family or personal history of long or short QT syndrome, Brugada syndrome or known history of QTc prolongation or Torsades de Pointes. Uncontrolled hypertension. Symptomatic or uncontrolled arrhythmia, including atrial fibrillation.
	Patients with ER-positive BC being treated with drugs recognised as strong inhibitors or inducers of the isoenzyme CYP3A which cannot be discontinued at least 7 days prior to planned treatment with ribociclib.
	Concomitant medication(s) with a known risk to prolong the QT interval and/or known to cause Torsades de Pointes that cannot be discontinued or replaced by a safe alternative medication.
	Pregnant or breastfeeding female patients, or patients who are planning to become pregnant.
	History of (non-infectious) interstitial lung disease (ILD)/pneumonitis that required steroids, has current ILD/pneumonitis, or where suspected ILD/pneumonitis cannot be ruled out by imaging at screening.
	Lung-specific intercurrent clinically significant illnesses, including, but not limited to, any underlying pulmonary disorder (eg, pulmonary emboli within 3 months of the study enrolment, severe asthma, severe COPD, restrictive lung disease, pleural effusion, etc).
	Any autoimmune, connective tissue or inflammatory disorders (eg, rheumatoid arthritis, Sjögren’s syndrome, sarcoidosis, etc.) with documented or suspected pulmonary involvement at the time of screening. Full details of the disorder should be recorded in the eCRF for patients who are included in the study.
	Prior pneumonectomy.
	History of positive testing for HIV or known AIDS.
	Acute or chronic infection with hepatitis B or C virus.
	Any impairment of gastrointestinal function or disease that may significantly alter the absorption of the study drugs (eg, uncontrolled ulcerative diseases, uncontrolled nausea, vomiting, diarrhoea, malabsorption syndrome or small bowel resection).
	Receipt of live, attenuated vaccine within 30 days prior to the first dose of T-DXd. Note: patients, if enrolled, should not receive a live vaccine during the study and up to 30 days after the last dose of study drug.
	Any psychological, including substance abuse, familial, sociological or geographical condition potentially hampering compliance with the study protocol and follow-up schedule; those conditions should be discussed with the patient before registration in the trial.
	Allergic reactions or hypersensitivity to the study drugs or other monoclonal antibodies.
	Administration of other experimental drugs, either concomitantly or during the past 30 days before treatment initiation.
	Pretreatment axillary surgery.
	Recent major surgery (within 4 weeks from the start of study treatment) or anticipated need for major surgery during the study.
	A pleural effusion, ascites or pericardial effusion that requires drainage, peritoneal shunt or Cell-free and Concentrated Ascites Reinfusion Therapy.

ALT, alanine aminotransferase; aPTT, activated partial thromboplastin time; AST, aspartate aminotransferase; BC, breast cancer; CISH, Chromogenic In Situ Hybridization; DCIS, ductal carcinoma in situ; ECOG, Eastern Cooperative Oncology Group; eCRF, electronic case report form; FISH, Fluoroscence In Situ Hybridization; GCP, Good Clinical Practice; HER2, human epidermal growth factor receptor 2 ; ICH, International Council for Harmonisation; IHC, Immunohistochemistry; ILD, interstitial lung disease; INR, International Normalized Ratio; ISH, In Situ Hybridization; LVEF, left ventricular ejection fraction; PR, Progesterone Receptor; PT, prothrombin time; SISH, Silver-enhanced In Situ Hybridization; T-DXd, trastuzumab deruxtecan; ULN, upper limit of normal.

### Study treatment

T-DXd is administered intravenously 5.4 mg/kg on Day 1 every 21 days. TCHP is administered intravenously on Day 1 every 21 days as follows: docetaxel 75 mg/m^2^, carboplatin 5 AUC (area under curve) and H 8 mg/kg loading dose followed by 6 mg/kg, and P 840 mg loading dose followed by 420 mg. When clinically indicated, weekly paclitaxel (80 mg/m^2^) and carboplatin (1.5 AUC) can be used instead of tri-weekly docetaxel and carboplatin.

After the first three cycles, further treatment is dictated by the intrinsic molecular subtype and the radiologic response to treatment according to contrast-enhanced MRI (figure 1). DD EC is given as epirubicin 90 mg/m^2^ intravenously on Day 1 every 14 days for four cycles and cyclophosphamide 600 mg/m^2^ intravenously on Day 1 every 14 days for four cycles. Ribociclib is taken orally at 600 mg per day for 3 weeks, followed by a 1-week pause, for 8 weeks. Letrozole is taken orally at 2.5 mg per day continuously for 8 weeks.

### Study interventions

**Figure 2:**
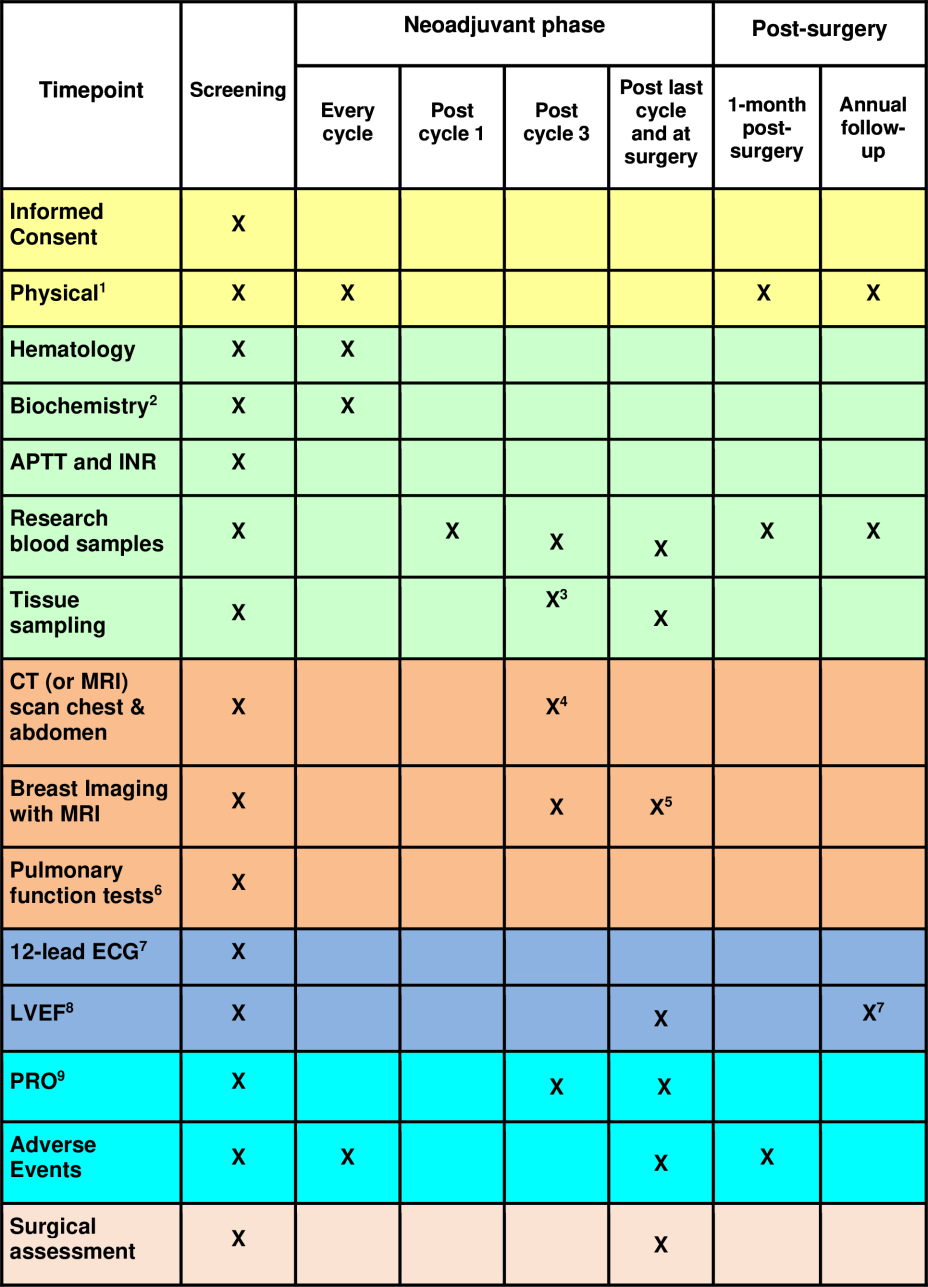
List of study interventions before the start of the treatment, during the treatment and at the follow-up phase. ^1^Eastern Cooperative Oncology Group performance status, symptoms, vital signs, oxygen saturation and assessment of primary tumour and regional lymph nodes. ^2^Haematology, serum chemistry, N-terminal pro brain natriuretic peptide, troponin T. Also FSH, oestradiol (sensitive assay), antimüllerian hormone and pregnancy test 7 days before the start of the study therapy for premenopausal and perimenopausal patients. ^3^Core biopsy, postcycle 3, is only performed on patients with radiologically residual cancer measuring at least 10 mm according to MRI. ^4^Chest CT scan only for patients who will continue with three additional cycles with trastuzumab deruxtecan. ^5^Breast MRI is optional postcycle. ^6^Pulmonary function tests, including DLCO, are recommended but not obligatory. ^7^ECG is done at baseline and thereafter if clinically indicated. For patients treated with ribociclib, it is performed per local guidelines. ^8^Left Ventricular Ejection Fraction (LVEF) is obtained at baseline, postcycle 6, during the adjuvant phase according to local practice, 1 year postoperatively and 5 years postoperatively. ^9^Patient-reported outcomes (PROs) include the European Organisation for Research and Treatment of Cancer (EORTC) Quality of Life Instrument QLQ-C30 and the EORTC QLQ-BR23 instrument. PROs are obtained at baseline, after three cycles of NAT, at the end of NAT, 1 year after surgery and 5 years after surgery. aPTT: activated partial thromboplastin time; DLCO: Diffusing Capacity of the Lungs for Carbon Monoxide; INR: international normalized ratio; NAT: neoadjuvant therapy; QLQ-BR23: Breast cancer Quality of Life questionnaire; QLQ-C30: Core Quality of Life questionnaire.

A summary of all trial-related procedures is provided in figure 2. All patients are followed during the neoadjuvant phase with office visits for physical examination, documentation of AEs and routine blood work before every treatment cycle.

Up to five (at least 4) core biopsies (14G needle) are obtained from all patients within 2 weeks before the start and 16±2 days after cycle 3 (only for patients that have a radiologically residual tumour measuring at least 10 mm according to MRI), respectively, and a similar amount of tissue is obtained from the tumour sample in situ immediately after removal by the surgeon from all patients.

PAM50 (Prosigna) is performed after enrolment and before the start of the treatment, using RNA extracted from the initial diagnostic core biopsy material.

Blood samples (2×10 mL) are obtained at several timepoints: baseline, postcycle 1, 3 and 6, just before surgery, just before the start of adjuvant therapy, annually at follow-up visits and at the time of relapse.

Contrast-enhanced MRI of the breast is performed on all patients prior to the start of the treatment and after three treatment cycles. MRI after cycle 6 is recommended if clinically indicated.

Patient-reported outcomes using the European Organisation for Research and Treatment of Cancer (EORTC) QLQ-C30 (Core Quality of Life questionnaire) and the EORTC QLQ-BR23 (Breast cancer Quality of Life questionnaire) instruments are obtained at baseline after three cycles of neoadjuvant treatment, at the end of neoadjuvant treatment and at 1 and 5 years after surgery.

### Study endpoints

The primary endpoint of ARIADNE is the locally assessed rate of pCR in the molecularly HER2-enriched population, defined as ypT0/Tis, ypN0, as determined from the surgical specimen by a pathologist blinded to treatment assignment (ITT analysis). Key secondary endpoints are the locally assessed rate of pCR, defined as ypT0/Tis, ypN0, in the two patient groups of the initial randomisation of TCHP versus T-DXd, regardless of administered therapy after cycle 3; event-free survival, defined as time from randomisation to BC relapse, contralateral BC, other malignant neoplasms or death from any cause, for each molecular group, including the comparison of TCHP versus T-DXd in HER2-enriched patients and in the two patient groups of the initial randomisation of TCHP versus T-DXd and the discovery of biomarkers of response/resistance to administered neoadjuvant therapy at the protein, RNA and DNA levels in both tumour tissue and blood/plasma. A full list of all study endpoints is provided in table 3.

**Table 3:** Study endpoints of the ARIADNE trial

Primary endpoint	Locally assessed rate of pCR at the molecularly HER2-enriched population, defined as ypT0/Tis, ypN0, as determined from the surgical specimen by a pathologist blinded to treatment assignment (ITT analysis).
Key secondary endpoints	Locally assessed rate of pCR, defined as ypT0/Tis, ypN0, at the two patient groups of the initial randomisation of TCHP versus T-DXd, regardless of administered therapy after cycle 3.
	EFS, defined as time from randomisation to BC relapse, contralateral BC, other malignant neoplasms or death from any cause, for each molecular group, including the comparison of TCHP versus T-DXd in HER2-enriched patients, and at the two patient groups of the initial randomisation of TCHP versus T-DXd.
	Discovery of biomarkers of response/resistance to administered neoadjuvant therapy at the protein, RNA and DNA levels in both tumour tissue and blood/plasma.
Other secondary endpoints	OS, defined as time from randomisation to death from any cause, for each molecular group, including the comparison of TCHP versus T-DXd in HER2-enriched patients, and at the two patient groups of the initial randomisation of TCHP versus T-DXd.
	DRFS, defined as time from randomisation to distant metastases or death from any cause, for each molecular group, including the comparison of TCHP versus T-DXd in HER2-enriched patients, and at the two patient groups of the initial randomisation of TCHP versus T-DXd.
	Locally assessed rate of pCR at ER-positive and luminal subgroups.
	Locally assessed rate of pCR at ER-negative and luminal subgroups, at the basal-like subgroup and the normal-like subgroup.
	Locally assessed rate of pCR at all molecular subgroups in the evaluable population, as defined in the protocol.
	Rates of radiologic complete response after three courses of either standard therapy or T-DXd.
	Rates of complete radiologic response, for each molecular group, including the comparison of TCHP versus T-DXd in HER2-enriched patients, and at the two patient groups of the initial randomisation of TCHP versus T-DXd.
	Pathologic response according to RCB class for each molecular group, including the comparison of TCHP versus T-DXd in HER2-enriched patients, and at the two patient groups of the initial randomisation of TCHP versus T-DXd.
	Rate of BCS, for each molecular group, including the comparison of TCHP versus T-DXd in HER2-enriched patients, and at the two patient groups of the initial randomisation of TCHP versus T-DXd.
	Rate of de-escalation of breast surgery (conversion from mastectomy to BCS or de-escalation of complexity from an oncoplastic breast-conserving procedure to simple wide local excisions) for each molecular group, including the comparison of TCHP versus T-DXd in HER2-enriched patients, and at the two patient groups of the initial randomisation of TCHP versus T-DXd.
	Rate of SLND, for each molecular group, including the comparison of TCHP versus T-DXd in HER2-enriched patients, and at the two patient groups of the initial randomisation of TCHP versus T-DXd.
	Rate of de-escalation of axillary surgery (conversion from axillary lymph node dissection to either targeted axillary dissection or SLND) for each molecular group, including the comparison of TCHP versus T-DXd in HER2-enriched patients, and at the two patient groups of the initial randomisation of TCHP versus T-DXd.
	Rate of sentinel lymph node detection, targeted axillary dissection success and false-negative rates of these procedures in initially node-positive patients for each molecular group, including the comparison of TCHP versus T-DXd in HER2-enriched patients, and at the two patient groups of the initial randomisation of TCHP versus T-DXd.
	Frequency and grade of AEs according to NCI CTCAE V.5.0 and rate of discontinuation due to toxicity.
	PROs, including HRQoL scores (EORTC QoL instrument (QLQ-C30); EEORTC breast cancer module (EORTC QLQ-BR23)).
	Exploratory analyses of clinicopathological characteristics as predictors for response.

AE, adverse event; BC, breast cancer; BCS, breast conserving surgery; CTCAE, Common Terminology Criteria for Adverse Events; DRFS, distant relapse-free survival; EFS, event-free survival; EORTC, European Organisation for Research and Treatment of Cancer ; ER, oestrogen receptor; HER2, human epidermal growth factor receptor 2; HRQoL, health-related quality of life; ITT, intention-to-treat; NCI, National cancer institute; OS, overall survival; pCR, pathologic complete response; PRO, patient-reported outcome; QLQ-BR23, Breast cancer Quality of Life questionnaire; QLQ-C30, Core Quality of Life questionnaire; QoL, quality of Life; RCB, residual cancer burden; SLND, sentinel lymph node dissection; TCHP, docetaxel, carboplatin, trastuzumab, pertuzumab; T-DXd, trastuzumab deruxtecan.

### Sample size calculation and statistical analysis

The ARIADNE study is designed to test whether neoadjuvant T-DXd treatment improves the pCR rate in patients with HER2-enriched BC. The null hypothesis for the primary endpoint is that the true pCR in the two treatment groups is the same. The alternative hypothesis is that the true pCR is different between treatment arms.

Prior experience with T-DM1 supports pCR rates of approximately 45% in molecularly unselected HER2-positive BC,^12^ which reaches 62% in HER2-enriched patients.^23^ A pCR rate of 70% is assumed for TCHP in HER2-enriched patients, based on prior results from the KRISTINE trial.^23^ At the time of study design, no data on neoadjuvant T-DXd had been published, but based on its significant activity in metastatic disease compared with T-DM1, higher pCR rates of 84% are expected for HER2-enriched BC.

To detect such a difference with 80% statistical power and using a two-sided type I error of 0.1, the study needs to include 218 molecularly HER2-enriched patients. Approximately 60% of all clinically HER2-positive BC is HER2-enriched.^17^ For a population of 218 patients with HER2-enriched BC to be treated with either standard therapy or T-DXd, as described above, at least 363 patients will need to be enrolled. Of the 145 remaining patients, approximately 109 patients are expected to be molecularly luminal ER-positive (up to 30% of all HER2-positive patients) and 36 normal-like, basal-like or ER-negative and luminal. Drop-out in previous PREDIX trials has been around 2% in total; consequently, a total of 370 patients will need to be included in the study.

The primary endpoint is the estimation and comparison of the pCR rates, defined as ypT0/TisN0 from the surgical specimen, as assessed locally. Counts and percentages of pCR will be reported per treatment arm, along with the associated 95% Clopper-Pearson CI. The Cochran-Mantel-Haenszel test, stratified by the stratification factors at randomisation, will be used to formally compare the pCR rates between the two arms. The analysis will be performed in the ITT population, which includes all patients who are randomised according to the treatment groups from initial randomisation. Patients with no pCR assessment will be considered as non-pCR. Sensitivity analysis using the evaluable population will also be conducted to assess the robustness of the results. As an exploratory analysis, multivariate logistic regression analysis will be used to assess covariates that are associated with pCR, estimating the OR and the associated 95% CI between subgroups of interest. Time-to-event endpoints will be analysed according to the Kaplan-Meier method in the ITT population. Kaplan-Meier survival curves and the median estimation will be reported, along with the associated 95% CI calculated using the SE derived from Greenwood’s formula. As an exploratory analysis, univariate Cox proportional hazards models will be used to calculate the risk reduction. HR with 95% CI will be reported to evaluate the difference between treatment arms and between subgroups of interest.

Within ARIADNE, a breast surgery study is predefined in the protocol. Intended and actual breast surgery will be documented. The difference in the optimal resection volume at baseline and postneoadjuvant treatment will be used as a surrogate of the possibilities for surgical de-escalation in the breast in each patient. Surgical de-escalation for the study will be expressed as the numerical difference of the optimal resection ratio before and after neoadjuvant therapy. Actual de-escalation strategies will also assess the shift from mastectomy to breast conserving surgery (BCS), and from oncoplastic BCS to standard BCS. Surgical de-escalation of the axilla will be expressed as the shift from axillary lymph node dissection to targeted axillary dissection or sentinel lymph node dissection for initially node positive patients who have converted to ycN0.

### Patient and public involvement

There was no patient or public involvement during the design of the trial and drafting of the protocol.

### Ethical considerations and dissemination

The responsible investigator will ensure that this study is conducted in accordance with the Declaration of Helsinki (including the Tokyo, Venice, Hong Kong, Somerset West and Edinburgh amendments) or the national laws and regulations, whichever provides the greater protection for the patient. In addition, the study will comply with the General Data Protection Regulation. The protocol has been written, and the study will be conducted according to the International Council for Harmonisation (ICH) Harmonised Tripartite Guideline for Good Clinical Practice. The protocol will be approved by the local ethics committee of each participating centre before the first patient can be included in that centre. There was no patient or public involvement during the design of the trial and drafting of the protocol.

The application for approval of the ARIADNE trial was submitted to the European Clinical Trials Information System portal on 21 December 2022. The study was approved by the Swedish Medical Products Agency (Läkemedelsverket) for Part I and the Swedish Ethical Review Authority (Etikprövningsmyndigheten) for Part II on 19 April 2023 (dnr 5.1.1-2022-105383). Stockholm Medical Biobank approved the trial on 20 April 2023. The application was submitted to the Norwegian Ethics Committee for Clinical Trials on Medicinal Products and Medical Devices on 7 December 2023, and the trial was approved on 20 February 2024. The trial protocol has been approved by review boards at all participating centres. Applications for ethical approval in Belgium, the Netherlands and Italy are ongoing.

Substantial amendments, that is, those likely to have an impact on the safety of the trial subjects, change the interpretation of the scientific documents in the conduct of the trial or are otherwise significant, must be reviewed and approved by the competent authority and ethics committee. All changes will be reflected in the patient-informed consent form.

Precautions are taken to preserve confidentiality and to prevent genetic data from being linked to the identity of the patient. In exceptional circumstances, however, certain individuals may access both the genetic data and the personal identifiers of a patient. Also, the regulatory authority may require access to the relevant files, though the patient’s medical information and the genetic files would remain physically separate.

It is our intention to publish the results of the study in a scientific journal. The companies supporting the trial financially have no influence on the contents. The Vancouver Declaration will be followed in all publications based on this trial. The study results will be submitted to the EU database within 1 year after the end of the CT, in accordance with the CTs Regulation Article 37.

### Study financing

ARIADNE is financed by the Swedish Research Council (Vetenskapsrådet), the Swedish Cancer Society (Cancerfonden), AstraZeneca (Cambridge, UK) and Novartis (Basel, Switzerland). T-DXd is provided free of cost by AstraZeneca. Ribociclib is provided free of cost by Novartis. Veracyte (South San Francisco, California, USA) provides kits for Prosigna at a reduced price. Funders have no role in study design, collection, management, analysis, interpretation of data, writing of the report or the decision to submit the report for publication, although they have read and provided comments on the study protocol.

## Supplementary material

10.1136/bmjopen-2025-102626 online supplemental file 1
