# Supplementary material for: Randomised trial of trastuzumab deruxtecan and biology-driven selection of neoadjuvant treatment for HER2-positive breast cancer: a study protocol of ARIADNE
Source: BMJ Open. 2025 Aug 27;15(8):e102626. doi: 10.1136/bmjopen-2025-102626 (PMC12410627; doi:10.1136/bmjopen-2025-102626)
Supplement: Supplementary file 2 [file bmjopen-2025-102626.reviewer_comments.pdf]

PEER REVIEW HISTORY

BMJ Open publishes all reviews undertaken for accepted manuscripts. Reviewers are asked to complete a checklist review form and are provided with free text boxes to elaborate on their assessment. These free text comments are reproduced below.

ARTICLE DETAILS

Title (Provisional)

Study protocol of ARIADNE: A Randomized Trial of Trastuzumab Deruxtecan and Biology-Driven Selection of Neoadjuvant Treatment for HER2-positive breast cancer

Authors

Matikas, Alexios; Naume, Bjørn; Wildiers, Hans; Sonke, Gabe; Dieci, Maria Vittoria; Karakatsanis, Andreas; Barnekow, Elin; Kessler, Luisa Edman; Einbeigi, Zakaria; Nearchou, Andreas; Engebraaten, Olav; Porojnicu, Alina; Soland, Mari Hiorth; Mannsåker, Bård; Raj, Sunil X.; Blix, Egil Støre; Nordstrand, Cecilie Soma; Vernieri, Claudio; Punie, Kevin; Sotiriou, Christos; Zouzos, Athanasios; Hellström, Mats; Hartman, Johan; Foukakis, Theodoros; Andersson, Anne; Villacampa, Guillermo; Bergh, Jonas; Lambertini , Matteo; Killander , Fredrika; Linderholm, Barbro; Schiza, Aglaia; Valachis, Antonis

---

VERSION 1 - REVIEW

**Reviewer**	**1**
**Name**	**Zhou, Jichun**
**Affiliation**	**Zhejiang University School of Medicine Sir Run Run Shaw Hospital, Surgical Oncology**
**Date**	**14-Jul-2025**
**COI**	**None**

The ARIADNE trial is a timely, hypothesis-driven exploration of precision neoadjuvant therapy in HER2-positive breast cancer. Its strengths lie in adaptive treatment stratification and translational ambition. Addressing operational challenges, ensuring biomarker consistency, and expanding endpoints to include long-term survival （EFS and OS） will further strengthen its impact. If successful, this trial could redefine neoadjuvant paradigms, balancing efficacy and de-escalation in molecularly defined subpopulations.

VERSION 1 - AUTHOR RESPONSE

Reviewer #1 (Comments to the Author):

The ARIADNE trial is a timely, hypothesis-driven exploration of precision neoadjuvant therapy in HER2-positive breast cancer. Its strengths lie in adaptive treatment stratification and translational ambition. Addressing operational challenges, ensuring biomarker consistency, and expanding endpoints to include long-term survival （EFS and OS）will further strengthen its impact. If successful, this trial could redefine neoadjuvant paradigms, balancing efficacy and de-escalation in molecularly defined subpopulations.

We thank the reviewer for their time and effort in reviewing the study protocol and for the positive comments.
